# Formation mechanisms of Fe_3−x_Sn_x_O_4_ by a chemical vapor transport (CVT) process

**DOI:** 10.1038/srep43463

**Published:** 2017-03-06

**Authors:** Zijian Su, Yuanbo Zhang, Bingbing Liu, Yingming Chen, Guanghui Li, Tao Jiang

**Affiliations:** 1 School of Minerals Processing and Bioengineering, Central South University, Changsha 410083, China

## Abstract

Our former study reported that Fe-Sn spinel (Fe_3−x_Sn_x_O_4_) was easily formed when SnO_2_ and Fe_3_O_4_ were roasted under CO-CO_2_ atmosphere at 900–1100 °C. However, the formation procedure is still unclear and there is a lack of theoretical research on the formation mechanism of the Fe-Sn spinel. In this work, the reaction mechanisms between SnO_2_ and Fe_3_O_4_ under CO-CO_2_ atmosphere were determined using XRD, VSM, SEM-EDS, XPS, etc. The results indicated that the formation of Fe_3−x_Sn_x_O_4_ could be divided into four steps: reduction of SnO_2_ to solid phase SnO, volatilization of gaseous SnO, adsorption of gaseous SnO on the surface of Fe_3_O_4_, and redox reaction between SnO and Fe_3_O_4_. During the roasting process, part of Fe^3+^ in Fe_3_O_4_ was reduced to Fe^2+^ by gaseous SnO, and meanwhile Sn^2+^ was oxidized to Sn^4+^ and entered into Fe_3−x_Sn_x_O_4_. The reaction between SnO_2_ and Fe_3_O_4_ could be summarized as Fe_3_O_4_ + xSnO_(g)_ → Fe_3−x_Sn_x_O_4_ (x = 0–1.0).

Binary and ternary metal oxides of tin (such as SnO, SnO_2_, Zn_2_SnO_4_, CaSnO_3_, BaSnO_3_, etc) have recently used as highly suitable semiconductor materials, which can be applied in electronic industrials^1^,^2^,^3^,^4^,^5^,^6^,^7^. For instance, tin-doped spinel (Fe_3−x_Sn_x_O_4_, x = 0~1.0) has been widely used as ferrimagnetic materials, electrical transformer cores, gas-detector sensors, heterogeneous catalysts and magnetic memory devices^8^,^9^,^10^,^11^,^12^. Previous synthetic methods for Sn-doped spinel were solid-state reactions, which required the synthesis temperature above 1300 °C and reaction time more than 10 hours^10^,^11^,^12^. However, excessively high temperature accelerated the formation of liquid phase, which resulted in a large grain size of the products. In order to obtain Fe-Sn spinel nanoparticles with regular shape and unique physical-chemical property, co-precipitation and precipitation exchange methods are commonly applied in the laboratory researches^10^,^11^,^12^,^13^. Aqueous solutions of iron (III) and tin (II) chlorines or nitrates were first prepared and mixed as a stoichiometric ratio, followed by adding NH_3_·H_2_O or NaOH solutions into the mixed solution to adjust the pH value. The precipitate products were filtrated and washed repeatedly, and then they were dried or roasted at low temperatures of 200–500 °C. However, due to the very low productivity most of those methods are only in a laboratory bench scale.

Our previous studies indicated that Fe-Sn spinel (Fe_3−x_Sn_x_O_4_), Ca_2_SnO_4_, CaSnO_3_, Na_2_SnO_3_ and SnSiO_3_ were much easily formed under CO-CO_2_ atmospheres^14^,^15^,^16^,^17^,^18^,^19^. It was reported that tin was inevitably volatilized as gaseous SnO when SnO_2_ was roasted at 900–1100 °C under different CO-CO_2_ atmospheres^20^,^21^. What’s more, the optimal conditions for the formation of Fe_3−x_Sn_x_O_4_ and tin volatilization were consistent based on a large number of experiments^14^,^20^. The intrinsic relationships between those processes need to be further investigated.

A chemical vapor-transport (CVT) method is commonly used for obtaining high-quality single crystals that are difficult or even impossible to be prepared by other methods^22^,^23^,^24^,^25^. The CVT method was successfully applied for synthesizing various inorganic compounds and separating some rare metals and rare earths^26^,^27^,^28^,^29^. As an important preparative method in solid state chemistry field, a considerable amount of works were focused on the homogeneous gas-phase equilibria, the temperature dependency of heterogeneous reactions, the crystallization controlling conditions, and so on. And the key problem of the CVT process was to strictly control the vaporization conditions of the volatile substances. As well-known, SnO_2_ has high melting point and boiling point, so solid state reactions between SnO_2_ and iron oxides are difficult to proceed. However, SnO is easily volatilized at the temperature above 900 °C, which is an excellent volatile substance for the CVT process.

Therefore, the Fe-Sn spinel (Fe_3−x_Sn_x_O_4_) was prepared from SnO_2_ and Fe_3_O_4_ by a CVT process under CO-CO_2_ atmosphere. The major objectives of this research were: (1) to investigate the formation mechanisms of Fe_3−x_Sn_x_O_4_ by a CVT process under 15 vol% CO/(CO + CO_2_) at 950 °C; (2) to reveal the effect of gaseous SnO as an intermediate volatile substances on the formation of Fe_3−x_Sn_x_O_4_; (3) to determine the redox reactions between gaseous SnO and Fe_3_O_4_ by using XRD, VSM, XPS, SEM-EDS, etc.

## Results

### Determination of the phase composition of the roasted samples with Fe_3_O_4_ and SnO_2_

Mixed samples (natural magnetite and cassiterite powders) were roasted at 950 °C under an atmosphere of 15 vol.% CO/(CO + CO_2_) for different time, and the roasted samples were then prepared for XRD, VSM, XPS and SEM-EDS analyses.

**Figure 1:**
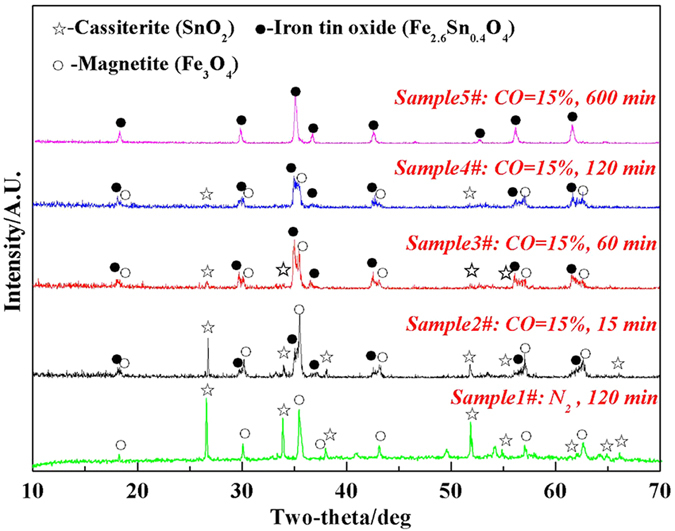
XRD patterns of the samples roasted at 950 °C for different time.

Figure 1 demonstrates the XRD patterns of the samples roasted at 950 °C for the time varying from 15 min to 600 min, and the sample roasted for 120 min in 100 vol.% N_2_ atmosphere was also measured. It can be seen from Fig. 1 that the main phase constitutions of the samples were magnetite, cassiterite and Fe-Sn spinel (Fe_2.6_Sn_0.4_O_4_) under 15 vol.% CO atmosphere. However, the diffraction peaks of Fe-Sn spinel remarkably enhanced as the roasting time prolonged, which indicated the gradual conversion of magnetite into Fe-Sn spinel. Interestingly, no diffraction peak of Fe-Sn spinel was found in the XRD pattern of the samples roasted under 100 vol.% N_2_ atmosphere, revealing that there was no reaction happening between Fe_3_O_4_ and SnO_2_ at 950 °C. Based on the above results, it is inferred that the CO-CO_2_ atmosphere plays an important role in the formation of Fe-Sn spinel.

**Figure 2:**
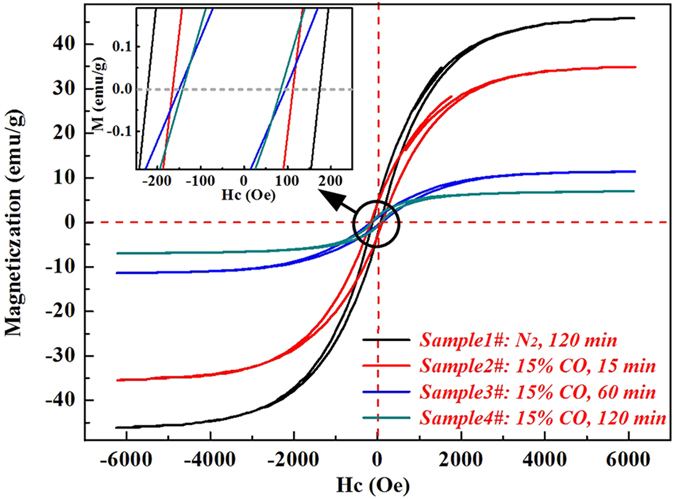
Magnetic hysteresis loops of the samples roasted at 950 °C.

In order to investigate the transformation process of Fe-Sn spinel, the magnetization hysteresis loops of the above-mentioned samples (**No. 1#~4#**) were studied by VSM at room temperature, and the results are displayed in Fig. 2. The results in Fig. 2 showed that the saturation magnetization (M_S_) of **Sample 1#** was about 47.6 emu/g, because the magnetite was stable when roasted under N_2_ atmosphere and there was no Fe-Sn spinel formed during the roasting. The Ms values of the samples (**2#~4#**) were much lower than that of **Sample 1#**. As the roasting time increased from 15 min to 120 min, the Ms value decreased obviously from 36.2 emu/g to 7.3 emu/g. In addition, it was observed from Fig. 2 that the coercivity field (the value of Hc when Ms is equal to zero) also decreased markedly with the increase of roasting time. Previous studies showed that Sn^4+^ could replace the Fe^3+^ in the magnetite to form Fe-Sn spinel, which resulted in a smaller hysteresis as well as the coercivity field^10^,^11^. As reported, Sn^4+^ could enter into the octahedral sublattice of magnetite, and then Fe-Sn spinel was easily formed under CO-CO_2_ atmosphere, which led to the decrease of saturation magnetization with the roasting time increasing.

**Figure 3:**
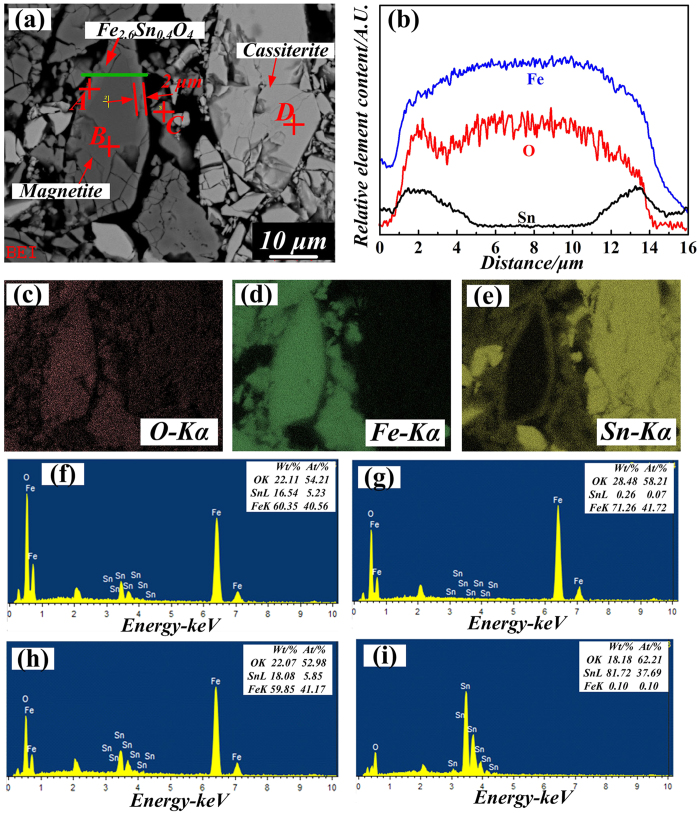
SEM-EDS analysis of roasted sample (**Sample 3#**) (**a**)-BS image; (**b**) the corresponding elements distribution along the green line in image (**a**); (**c**), (**d**) and (**e**)-corresponding elements’ area distribution images of O, Fe and Sn; (**f**), (**g**), (**h**) and (**i**) - EDS of spot A, B, C and D in image (**a**).

To analyze the element distribution and composition of the Fe-Sn spinel formed in the roasted sample (**Sample 3#**), the backscattered micrographs of Fe-Sn spinel and the corresponding elements’ area distribution images by SEM-EDS are shown in Fig. 3. As seen from Fig. 3a, the major phases in the sample were magnetite (Spot B), cassiterite (Spot D) and Fe-Sn spinel (Spot A and C). Moreover, the Fe/Sn atomic ratio of Spot A and Spot C was similar to the value of 2.6: 0.4, which was coincident with the result presented in Fig. 1. In addition, it was amazing to find that the Fe-Sn spinel displayed as a thin layer and enwrapped the magnetite compactly. Based on the results in Fig. 3b and e, the corresponding Sn element’s distribution indicated that Fe-Sn spinel was formed on the outside surface of magnetite particle. Obvious elemental gradient of Sn from the outside surface to the inner was observed in Fig. 3b, and there was almost no Sn element existing in the center part of the magnetite particle. However, Fe element was not found on the surface of cassiterite as shown in Fig. 3i. Therefore, there existed the mass transfer of Sn from SnO_2_ to Fe_3_O_4_, and the formation mechanism would be further researched.

**Figure 4:**
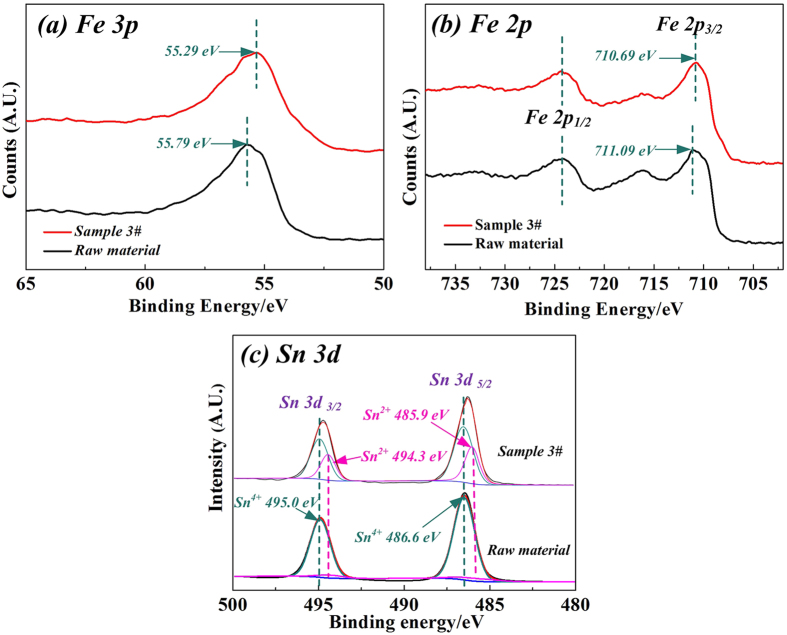
The XPS spectra of Fe 3p, Fe 2p and Sn 3d of **Raw Material** and **Sample 3#**.

X-ray photoelectron spectroscopy (XPS) was then applied to check the chemical state of the samples’ surfaces. The Fe 3p, Fe 2p and Sn 3d photoelectron spectra of the **Raw material** (magnetite and cassiterite powders were blended as mass ratio of 4:1) and **Sample 3#** are shown in Fig. 4. Based on the reported XPS studies of Fe 3p and Fe 2p, the photoelectron peaks of Fe are always associated with satellite peaks and background noise, which are complicated to distinguish definitely^30^,^31^. As shown in Fig. 4a and b, the binding energy of Fe 3p and Fe 2p_3/2_ in **Sample 3#** shifted obviously from 55.79 eV to 55.29 eV and 711.09 eV to 710.69 eV, respectively. The decrease of the Fe binding energy was attributed to the replacement of Fe^3+^ by Sn^4+^ in Fe_3_O_4_, and then the Fe^3+^ in Fe_3_O_4_ was partially converted into Fe^2+^ for the charge balance^8^,^9^,^10^,^11^. As observed from Fig. 4c, the Sn 3d photoelectron peak of the **Raw Material** was well matched with the peaks of pure SnO_2_ in the previous literatures^32^,^33^. However, the XPS photoelectron peak of Sn 3d in Fig. 4c can be resolved into Sn^2+^ and Sn^4+^. And both of Sn 3d 5/2 and Sn 3d 3/2 clearly showed two groups of Sn chemical bonding energies of 486.6 eV and 495.0 eV for Sn^4+^, and 494.3 eV and 485.9 eV for Sn^2+^ 32,33. Our previous studies on the reduction roasting of SnO_2_ have proved that there is no SnO existing in the roasted samples^14^,^20^,^21^. Therefore, the resolved peaks of Sn^2+^ (Fig. 4c) just displayed the electron deficiency state of Sn on the surface of **Sample 3#**, indicating that the intermediate product, SnO, would be crucial to the formation of Fe-Sn spinel.

### Reactions between Fe_3_O_4_ and gaseous SnO

As reported in our former studies, the main reactions of SnO_2_ roasted at 950 °C under 15 vol.% CO atmosphere were expressed as the following Eq. (1) and Eq. (2)^14^,^20^,^21^. Equation (2) was carried out rapidly and no SnO_(s)_ was found in the roasted samples.


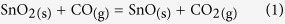



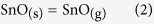


**Figure 5:**
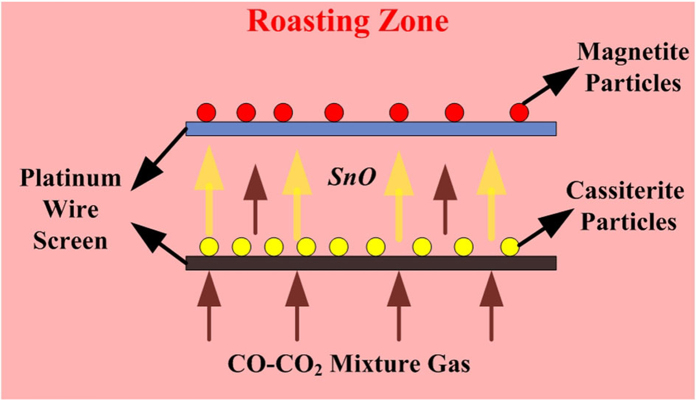
The schematic diagram for investigating the reactions between Fe_3_O_4_ and gaseous SnO.

In this section, the reaction between Fe_3_O_4_ and gaseous SnO was investigated and the schematic diagram of the experiment was shown in Fig. 5. A platinum wire screen was used to separate the cassiterite and magnetite particles, and then the samples were placed into an electrically-heated vertical-tube furnace and roasted at 950 °C for 60 min under 15 vol.% CO atmosphere. In this system, the solid-solid reactions between Fe_3_O_4_ and SnO_2_ were impossible to proceed, so that the effect of gaseous SnO on the formation of Fe_3−x_Sn_x_O_4_ could be investigated.

**Figure 6:**
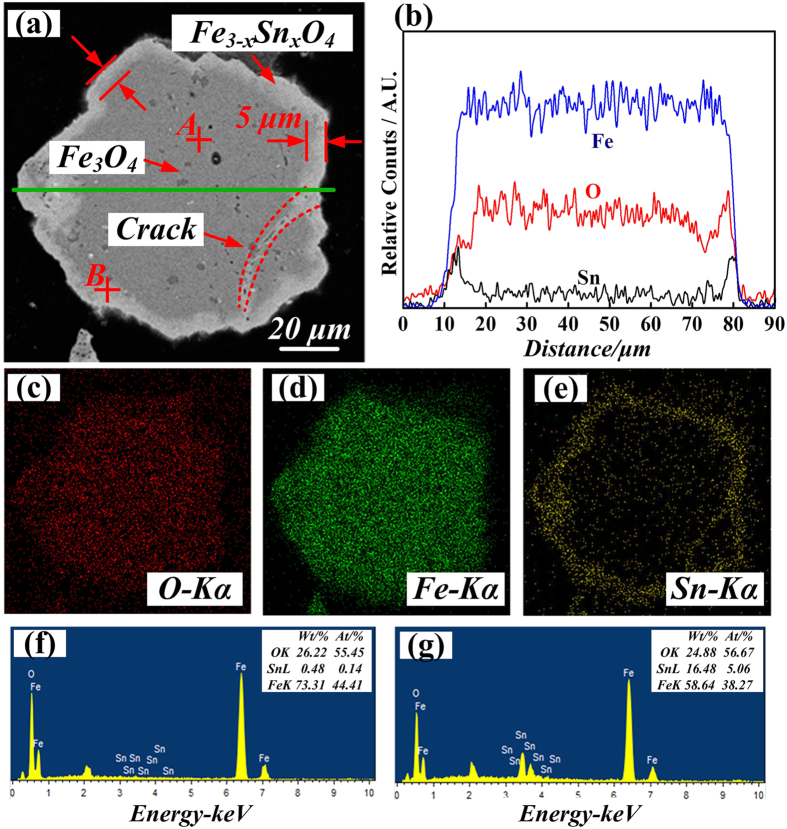
SEM-EDS analysis of products of Fe_3_O_4_ and SnO_(g)_ (**a**)-BS image; (**b**) the corresponding elements distribution along the green line in image (**a**); (**c**), (**d**) and (**e**)-corresponding elements’ area distribution images of O, Fe and Sn; (**f**) and (**g**)-EDS of point A and B in image (**a**).

The SEM-EDS analyses of the roasted magnetite particles are shown in Fig. 6. It was observed from Fig. 6 that Fe-Sn spinel layer with a thickness of about 5 μm was formed at the outer sphere of the magnetite particles. The microstructure of the roasted sample in Fig. 6 was similar to that in Fig. 3. The corresponding elemental distributions of Sn and Fe indicated that the reactions between Fe_3_O_4_ and gaseous SnO took place as a typically unreacted core model, so an obvious product layer was formed outside the magnetite particles. Occasionally, a crack throughout the magnetite particle was found in Fig. 6, and the enrichment of Sn element propagated along with the crack. The results further confirmed our inference that gaseous SnO was the vital medium for the mass transfer of Sn during the formation of Fe-Sn spinel. Gaseous SnO was a volatile substance, which played an important role in the CVT process.

### Formation mechanisms of Fe_3−x_Sn_x_O_4_

The above-mentioned results indicated that the Fe-Sn spinel was formed via the reactions between gaseous SnO and Fe_3_O_4_, and the reaction could be summarized as Fe_3_O_4_ + xSnO_(g)_ → Fe_3−x_Sn_x_O_4_ (x = 0–1.0). It was reported that the valence state of Sn in the Fe_3−x_Sn_x_O_4_ was + 4 8,9,10,11,12, and the redox reactions between gaseous SnO and FeOx were discussed in this section.

**Figure 7:**
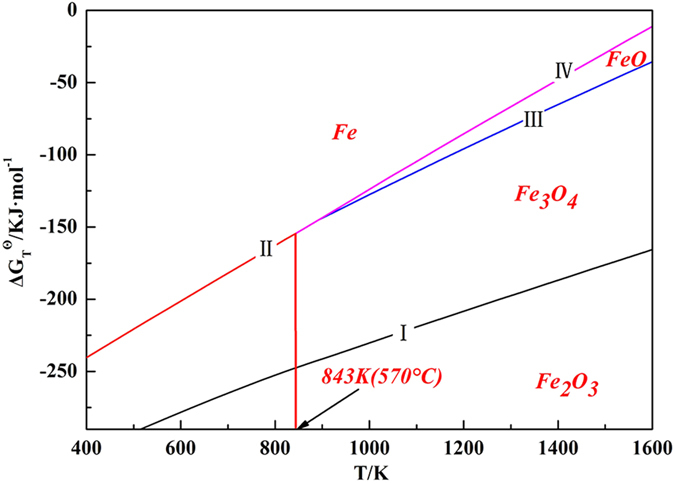
∆G^θ^-T relationship lines of the possible reactions between FeOx and gaseous SnO.

Below 570 °C, Fe_2_O_3_ is reduced as the stepwise order of Fe_2_O_3_ → Fe_3_O_4_ → Fe. When the temperature is higher than 570 °C, the reduction process would be Fe_2_O_3_ → Fe_3_O_4_ → FeO → Fe^19^,^20^,^21^. Thus, the possible chemical reactions between FeOx and gaseous SnO are given in Table 1, and the ∆G^θ^-T equations are also listed in Table 1 and Fig. 7.

**Figure 8:**
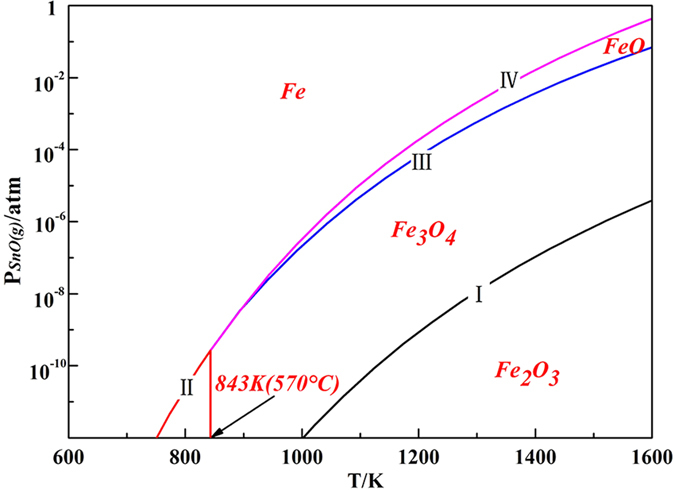
Gas-phase equilibrium diagram of FeOx under different SnO partial pressure.

The standard Gibbs free energy (∆G^θ^) change of the related reactions was calculated as follow:


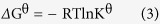


where R is the ideal gas constant (8.3144 J/mol·K), T is the temperature in kelvin (K), and K^θ^ is the standard equilibrium constant. In the reactions between gaseous SnO and FeOx, K^θ^ is equal to the reciprocal of the standard vapor pressure of gaseous SnO. Then, gas-phase equilibrium diagram of FeOx under different SnO partial pressure was calculated and plotted in Fig. 8.

Based on the results in Figs 7, 8 and Table 1, it was inferred that the reaction of Fe_3_O_4_ + xSnO → Fe_3−x_Sn_x_O_4_ happened and all the reactions between gaseous SnO and FeOx were obviously affected by the temperature and partial pressure of SnO. Fe_3_O_4_ was stable under 15 vol.% CO atmosphere at 950 °C, the SnO partial pressure was relatively low under this condition^20^,^21^, and the Sn in the Fe_3−x_Sn_x_O_4_ mainly existed as Sn^4+^ based on previous studies^8^,^9^,^10^. Then, the chemical formula of the spinel was calculated as the valence state balance, which were Fe_3_O_4_ of [Fe^2+^][Fe^3+^]_2_[O^2−^]_4_ and Fe_3−x_Sn_x_O_4_ of [Fe^2+^]_1+x_[Fe^3+^]_2−2x_[Sn^4+^]_x_[O^2−^]_4_. Hence, it was obvious that the valence state of Fe was remarkably affected by the Sn content in the Fe_3−x_Sn_x_O_4_. The more Sn^4+^ contained, the more Fe^2+^ was formed in the Fe_3−x_Sn_x_O_4_. In general, part of Fe^3+^ in Fe_3_O_4_ was reduced to Fe^2+^ by gaseous SnO, and Sn^2+^ was oxidized to Sn^4+^ and entered into Fe_3−x_Sn_x_O_4_. Thus, the mass transfer of Sn was conducted via a chemical vapor transport process.

**Figure 9:**
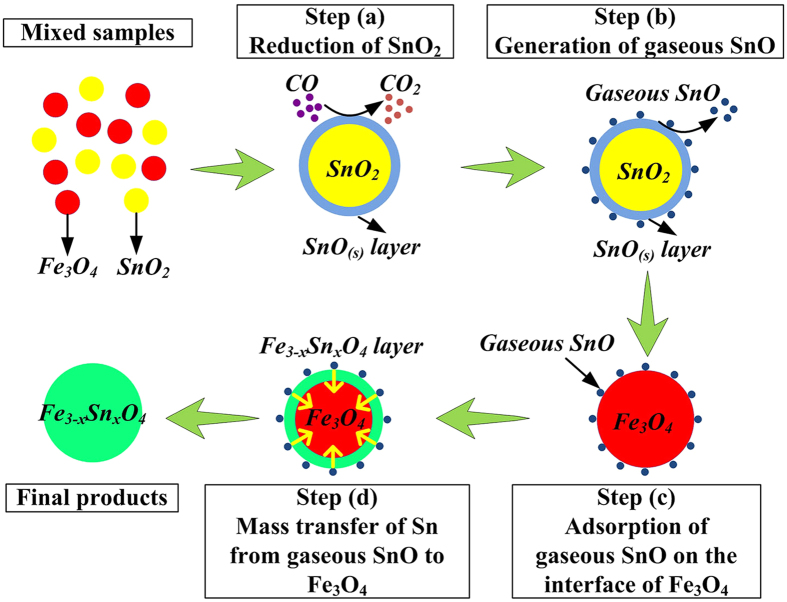
Schematic diagram for the formation of Fe_3−x_Sn_x_O_4_ by a CVT process.

The schematic diagram of the formation process of Fe_3−x_Sn_x_O_4_ by a CVT process is summarized in Fig. 9. Under the conditions of 15 vol.% CO atmosphere and roasting temperature of 950 °C, the reaction procedure between SnO_2_ and Fe_3_O_4_ could be described as follows: (a) SnO_2_ is reduced to solid phase SnO while Fe_3_O_4_ is stable under this condition; (b) SnO is volatilized as gaseous phase, and this process is much fast because no SnO_(s)_ is observed in the roasted samples^14^,^20^,^21^; (c) the gaseous SnO is adsorbed onto the interface of Fe_3_O_4_ particles; (d) the redox reaction between SnO and Fe_3_O_4_ takes place, resulting in the mass transfer of Sn from gaseous SnO into Fe_3_O_4_. There, the Fe_3−x_Sn_x_O_4_ is formed.

During this CVT process, it was found that formation of gaseous SnO was the critical step, which had obvious effect on the mass transfer of Sn and the redox reaction between SnO and Fe_3_O_4_.

## Conclusions

The formation mechanism of Fe_3−x_Sn_x_O_4_ from SnO_2_ and Fe_3_O_4_ by a CVT method was determined using XRD, VSM, SEM-EDS, XPS, etc. It is concluded that the formation of gaseous SnO under CO-CO_2_ atmosphere is the critical step, which has obvious effect on the the redox reactions between SnO and Fe_3_O_4_. The mass transfer of Sn from gaseous SnO into Fe_3_O_4_ was conducted via the chemical vapor transport process. The reactions between Fe_3_O_4_ and gaseous SnO, Fe_3_O_4_ + xSnO_(g)_ → Fe_3−x_Sn_x_O_4_ (x = 0–1.0), took place as a typical gas-solid unreacted core model. During the roasting process, part of Fe^3+^ in Fe_3_O_4_ was reduced to Fe^2+^ by gaseous SnO, and meanwhile Sn^2+^ was oxidized to Sn^4+^ and entered into Fe_3−x_Sn_x_O_4_.

## Method

Natural magnetite and cassiterite powders used in this study were the same as those given in our previous study^14^. The theoretical Fe_3_O_4_ and SnO_2_ contents of the samples were 98.7 wt.% and 98.5 wt.%, respectively. The purity of gases (CO, CO_2_ and N_2_) used in the tests was higher than 99.99 vol.%. All the roasting tests were conducted in a vertical-tube furnace. The natural magnetite and cassiterite powders were first blended at mass ratio of 4:1. Then, the mixed sample was put into a corundum crucible and roasted in the furnace. The CO/(CO + CO_2_) content was fixed at 15 vol.% and the roasting temperature was kept at 950 °C. The CO content refers to the CO volume concentration in the CO-CO_2_ mixed gas (i.e., CO/(CO + CO_2_)). After roasted for different time, the samples were taken out and quenched into liquid nitrogen rapidly. Finally, the cooled samples were used for analysis.

## Additional Information

**How to cite this article**: Su, Z. *et al*. Formation mechanisms of Fe_3−x_Sn_x_O_4_ by a chemical vapor transport (CVT) process. *Sci. Rep.* **7**, 43463; doi: 10.1038/srep43463 (2017).

**Publisher's note:** Springer Nature remains neutral with regard to jurisdictional claims in published maps and institutional affiliations.

## Supplementary Material

Supplementary Information

## Figures and Tables

**Table 1:** ∆G^θ^-T equations of the possible reactions between FeOx and gaseous SnO.

Eq.	Reactions	∆G^θ^-T (KJ/mol)
I	3Fe_2_O_3_ + SnO_(g)_ = 2Fe_3_O_4_ + SnO_2_	∆G^θ^ = 0.114 T–328.108
II	1/4Fe_3_O_4_ + SnO_(g)_ = 3/4Fe + SnO_2_	∆G^θ^ = 0.733 T–1269.481
III	Fe_3_O_4_ + SnO_(g)_ = 3FeO + SnO_2_	∆G^θ^ = 0.151 T–288.746
IV	FeO + SnO_(g)_ = Fe + SnO_2_	∆G^θ^ = 0.194 T–326.911

^*^Primary data of the substances mentioned above are obtained from Practical Thermodynamics Data Handbook of Inorganic Substances.
