# Supplementary material for: Formation mechanisms of Fe3−xSnxO4 by a chemical vapor transport (CVT) process
Source: Sci Rep. 2017 Mar 6;7:43463. doi: 10.1038/srep43463 (PMC5337948; doi:10.1038/srep43463)
Supplement: Supplementary Information [file srep43463-s1.doc]

**Supporting Information**

**Formation** **mechanisms of Fe_3-x_Sn_x_O_4_ by a** **chemical vapor transport (CVT) process**

Zijian Su, Yuanbo Zhang*, Bingbing Liu, Yingming Chen, Guanghui Li, Tao Jiang

**Fig. 1S**. Schematic diagram of experimental equipment for roasting

**Introductions for analysis method**

The mineral constituents of the roasted samples were identified by X-ray diffraction (XRD, D/max 2550PC, Japan Rigaku Co., Ltd) with the step of 0.005^o^ at 10^o^ min^-1^ in ranging from 10^o^ to 80^o^.

The changes of the sample magnetism were investigated by VSM (Vibration sample magnetometer, BHV-50HTI, Riken Keiki, in Japan).

XPS experimentation was performed with a Thermo Scientific ESCALAB 250Xi using an Al Ka X-ray source (1486.6 eV). A nonlinear least-square curve-fitting program (Avantage software 5.52) was used to deconvolve the XPS data.

The morphological change of the sample was detected using an environmental scanning electron microscope (ESEM; FEI QUANTA 200; FEI, Eindhoven, The Netherlands) equipped with an EDAX energy dispersive x-ray spectroscopy (EDS) detector (EDAX Inc., Mahwah, NJ).
